# Predicting the outcome of non-pharmacological treatment for patients with dementia-related mild cognitive impairment

**DOI:** 10.18632/aging.202270

**Published:** 2020-12-07

**Authors:** Yoshihito Shigihara, Hideyuki Hoshi, Jesús Poza, Víctor Rodríguez-González, Carlos Gómez, Takao Kanzawa

**Affiliations:** 1 Precision Medicine Centre, Hokuto Hospital, Obihiro 080-0833, Hokkaido, Japan; 2 MEG Centre, Kumagaya General Hospital, Kumagaya 360-8567, Saitama, Japan; 3 Biomedical Engineering Group, Higher Technical School of Telecommunications Engineering, University of Valladolid, Valladolid 47011, Castilla y León, Spain; 4 Centro de Investigación Biomédica en Red en Bioingeniería, Biomateriales y Nanomedicina, (CIBER-BBN), Valladolid 47011, Castilla y León, Spain; 5 Instituto de Investigación en Matemáticas (IMUVA), University of Valladolid, Valladolid 47011, Castilla y León, Spain; 6 The Dementia Center, Institute of Brain and Vessels Mihara Memorial Hospital, Isehara 372-0006, Gunma, Japan; 7 Isesaki Clinic, Gunma, Isehara 372-0056, Gunma, Japan

**Keywords:** dementia, mild cognitive impairment, non-pharmacological treatment, spectral parameters, power spectrum

## Abstract

Dementia is a progressive cognitive syndrome, with few effective pharmacological treatments that can slow its progress. Hence, non-pharmacological treatments (NPTs) play an important role in improving patient symptoms and quality of life. Designing the optimal personalised NPT strategy relies on objectively and quantitatively predicting the treatment outcome. Magnetoencephalography (MEG) findings can reflect the cognitive status of patients with dementia, and thus potentially predict NPT outcome. In the present study, 16 participants with cognitive impairment underwent NPT for several months. Their cognitive performance was evaluated based on the Mini-Mental State Examination and the Alzheimer's Disease Assessment Scale - Cognitive at the beginning and end of the NPT period, while resting-state brain activity was evaluated using MEG during the NPT period. Our results showed that the spectral properties of MEG signals predicted the changes in cognitive performance scores. High frequency oscillatory intensity at the right superior frontal gyrus medial segment, opercular part of the inferior frontal gyrus, triangular part of the inferior frontal gyrus, post central gyrus, and angular gyrus predicted the changes in cognitive performance scores. Thus, resting-state brain activity may be a powerful tool in designing personalised NPT.

## INTRODUCTION

Dementia is a syndrome characterised by progressive deterioration in cognitive functions (*e.g.*, memory, thinking, and behaviour) due to neurological disorders, such as Alzheimer's disease (AD) and stroke. Pharmacological treatments often fail to substantially improve the course of dementia [1, 2], and thus non-pharmacological treatments (NPTs) have played an important role in managing patient symptoms and improving their quality of life [2–5]. NPTs, which include various strategies, such as cognitive stimulation, occupational therapy, and music therapy [2, 5], induce neuroplasticity or change neural network efficiency, and thus ameliorate the symptoms of dementia [6, 7]. They are recommended as first line treatments and are especially effective in reducing the behavioural and psychological symptoms of dementia, which is one of the two categories of dementia symptoms [5, 8]. They are also effective in reducing cognitive impairment, the other category of dementia symptoms, which can be evaluated using instruments such as the Mini-Mental State Examination (MMSE) and Alzheimer's Disease Assessment Scale-Cognitive (ADAS-Cog) [9, 10]. However, evidence of the positive effects of NPTs is still preliminary [2–4, 8, 11], and considerable individual variability in NPT outcome has been noted; some patients respond positively, while others show little improvement. Moreover, whether the patient’s age or degree of dementia severity might predict treatment outcome is controversial [12]. Some studies also suggest that as yet uncharacterised factors, such as cognitive reserve, might modulate NPT outcome [6, 13]. Therefore, advances in devising new objective measurements that can predict NPT outcome would be very important because they could help determine the best NPT strategy for each patient.

In this regard, neuroimaging techniques can play an important role because they are able to monitor subtle changes in brain dynamics elicited by NPTs [14].

Magnetoencephalography (MEG) and electroencephalography (EEG) are non-invasive neuroimaging techniques that are sensitive to changes in neural activity induced by the neurodegenerative processes and progressive deterioration of synaptic activity associated with dementia [15–19]. Resting-state brain activity measured using MEG and EEG is represented as spontaneous neural oscillations that are characterised by their location, frequency, and intensity. There are three major characteristic alterations of the resting-state brain activity in dementia: (i) enhanced low frequency oscillatory activity accompanied by attenuated high frequency oscillatory activity, (ii) slowing down of the alpha peak frequency (so-called ‘shift-to-the-left of the alpha peak’), and (iii) less prominent alpha oscillations [20, 21]. Because MEG and EEG are sensitive to brain dynamics, they can predict the progress of cognitive decline [16, 22, 23] and reflect changes in neural activity caused by NPTs in patients with dementia or mild cognitive impairment (MCI) [14, 24]. Therefore, it can be hypothesised that neural oscillatory activity measured using MEG also has the potential to predict NPT outcome. To test this hypothesis, in the present study, 16 participants with cognitive impairments underwent NPTs for several months. Cognitive performance was assessed at the beginning and the end of the NPT period, whereas resting-state brain activity was evaluated using MEG during the NPT period. The spectral properties of MEG signals and the estimated regional neural oscillatory intensities were compared with the change in cognitive performance to evaluate whether resting-state brain activity was able to predict NPT outcome.

## RESULTS

### Cognitive assessment

**Figure 1:**
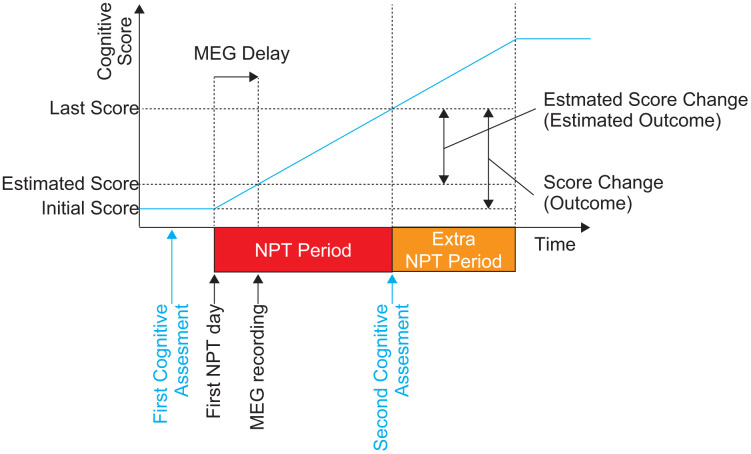
**The schematic description of the time course of the present study.** The diagram shows the order of the NPT, cognitive assessments, and MEG recording. Cognitive assessments (both MMSE-J and ADAS-J Cog) were conducted twice. The first assessment was performed before the first day of NPT. The NPT period was defined between the first day of NPT and day of the second cognitive assessments. MEG recording was conducted within the NPT period as early as possible. The blue line shows the hypothetical change in cognitive performance. NPT, non-pharmacological treatment; MMSE-J, Japanese version of the Mini-Mental State Examination; ADAS-J Cog, Japanese version of the Alzheimer's Disease Assessment Scale-Cognitive Subscale; MEG, magnetoencephalography.

The average ‘Initial Score’ for the Japanese version of the MMSE (MMSE-J) was 25.8 ± 2.9 (standard deviation, [SD]) (ranging from 20 to 30), and the ‘Last Score’ was 26.8 ± 2.7 (ranging from 22 to 30) (Figure 1). The score increased (*i.e.* the cognitive level improved) for nine participants, decreased for four participants, and remained unchanged for three participants. The mean score was significantly increased (1.0 ± 2.7, ranging from -3 to 7) at the group level (*p* = 0.034). Change in MMSE-J score (‘Outcome’) did not correlate with participant age (*r* = -0.09, *p* = 0.312), the length of the NPT period (*r* = 0.17, *p* = 0.300), the number of NPT sessions (*r* = 0.06, *p* = 0.428), or the frequency of the NPT sessions (*r* = -0.10, *p* = 0.360). The ‘Initial Score’ for the MMSE-J was negatively correlated with the change in MMSE-J score (‘Outcome’) (*r* = -0.47, *p* = 0.031). The average ‘Initial Score’ for the Japanese version of the ADAS-Cog (ADAS-J Cog) was 8.0 ± 3.4 (ranging from 0.8 to 13.7), and the ‘Last Score’ was 6.2 ± 3.3 (ranging from 0.4 to 12.7). The score decreased (*i.e.* the cognitive level improved) for 14 participants and increased for two participants. The average score was significantly decreased (-1.8 ± 2.1, ranging from -5.6 to 3.5) at the group level (*p* < 0.001). The length of the NPT period (*r* = 0.47, *p* = 0.023) and number of NPT sessions (*r* = 0.54, *p* = 0.015) were positively correlated with change in ADAS-J Cog score (‘Outcome’). In contrast, participant age (*r*= -0.35, *p* = 0.076), frequency of the intervention (*r* = 0.21, *p* = 0.129), and ‘Initial Score’ for the ADAS-J Cog (*r* = -0.37, *p* = 0.036) did not correlate with change in ADAS-J Cog score (‘Outcome’). The changes in MMSE-J and ADAS-J Cog scores were not correlated with each other (*r* = 0.28, *p* = 0.084). For two participants, the MMSE-J scores improved while the ADAS-J Cog scores worsened (both scores increased). For four participants, the MMSE-J scores worsened while the ADAS-J Cog scores improved (both scores decreased).

### Correlations between cognitive scores and spectral properties (sensor-level)

The ‘Estimated Scores’ of MMSE-J and ADAS-J Cog were 26.0 ± 2.9 (ranging from 20.6 to 30.0) and 7.8 ± 3.4 (ranging from 0.7 to 13.0). Their ‘Estimated Score changes’ were 6.2 ± 3.3 (ranging from -2.6 to 6.4) and -1.6 ± 1.8 (ranging from -5.6 to 2.4). None of the spectral parameters (median frequency [MF], individual alpha frequency [IAF] or the Shannon spectral entropy [SE]) showed significant correlations with the MMSE-J and ADAS-J Cog ‘Estimated Scores’ and ‘Last Scores’ (Figure 1). The ‘Estimated Score Change’ for both the MMSE-J and ADAS-J Cog (‘Estimated Outcome’) were negatively correlated with MF (*r* = -0.53, *p* = 0.030 for MMSE-J; *r* = -0.48, *p* = 0.017 for ADAS-J Cog) but not significantly correlated with either IAF or SE.

### Correlation between cognitive scores and regional oscillatory intensity (source-level)

**Figure 2:**
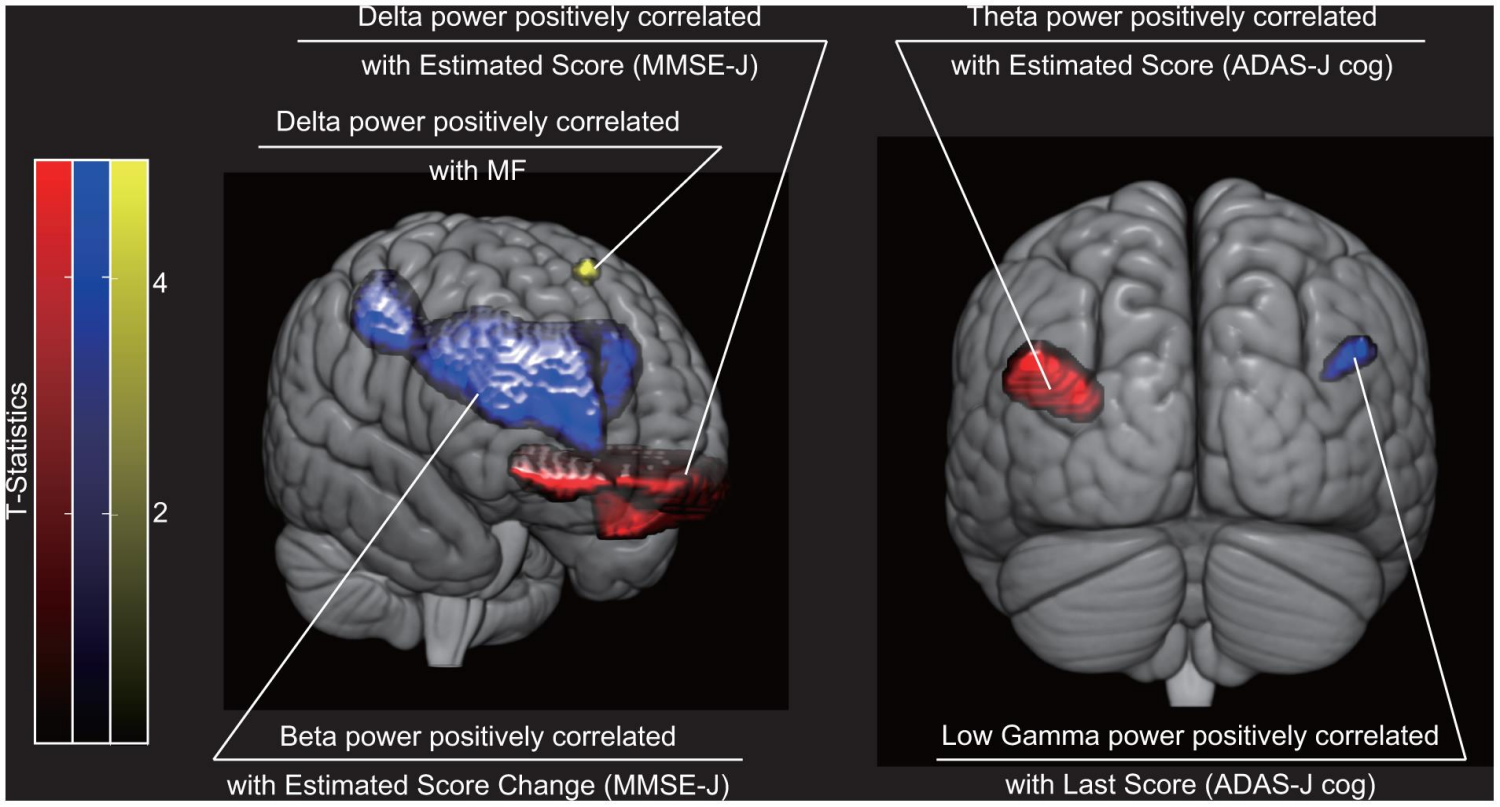
**Brain regions where cognitive sores were statistically significantly correlated with regional neural oscillatory intensity.** Red indicates brain regions where oscillatory intensity was correlated with cognitive scores on the recording day (*i.e.* ‘Estimated Scores’). Blue indicates brain regions where oscillatory intensity was correlated with cognitive score change (*i.e.* ‘Estimated Score Change’) or score at the end of the NPT period (*i.e.* ‘Last Score). Yellow indicates brain region where oscillatory intensity was correlated with a spectral property. The three dimensional images were created using MRIcroGL (https://www.mccauslandcenter.sc.edu/mricrogl/). NPT, non-pharmacological treatment; MF, median frequency; MMSE-J, Japanese version of the Mini-Mental State Examination; ADAS-J Cog, Japanese version of the Alzheimer's Disease Assessment Scale-Cognitive Subscale.

Regional oscillatory intensity at each frequency band was compared with cognitive scores in three ways: (i) with scores on the recording day (‘Estimated Scores’); (ii) with estimated changes in scores (‘Estimated Score Change’); and (iii) with scores at the end of the NPT period (‘Last Scores’). The MMSE-J ‘Estimated Score’ was positively correlated with delta intensity at the left anterior and right medial orbital gyrus, whereas the ADAS-J Cog ‘Estimated Score’ was positively correlated with theta intensity at the left angular gyrus (Table 1 and Figure 2). The MMSE-J ‘Estimated Score Change’ was positively correlated with the beta intensity at the right superior frontal gyrus medial segment, opercular part of the inferior frontal gyrus, triangular part of the inferior frontal gyrus, and post central gyrus. The ADAS-J Cog ‘Estimated Score Change’ score was not significantly correlated with oscillatory intensity. Finally, the ADAS-J Cog ‘Last Score’ was positively correlated with low-gamma intensity at the right angular gyrus. No other correlation was found.

**Table 1:** Correlation between source-level oscillatory intensity and other factors.

		**Frequency**	**Correlation**		**Cluster level**			**Peak level**			**Coordinates**				**Brain region**
					***p* (FWE)**	***kE***		***p* (FWE)**	***T***		**X**	**Y**	**Z**		
Estimated Score	MMSE	Delta	Positive		0.014	4501		0.002	7.384		-20	40	-8		Lt anterior orbital gyrus
								0.005	6.636		18	40	-8		Rt medial orbital gyrus
	ADAS	Theta	Positive		0.042	498		0.021	5.132		-38	-68	22		Lt angular gyrus
Estimated Score Change	MMSE	Beta	Positive		0.009	8591		0.021	5.316		14	42	22		Rt superior frontal gyrus medial segment
								0.036	4.921		40	18	26		Rt middle frontal gyrus
								0.039	4.861		48	-12	44		Rt post central gyrus
Last Score	ADAS	Low-gamma	Positive		0.048	61		0.045	4.636		46	-70	30		Rt angular gyrus
MF		Delta	Positive		0.049	22		0.037	4.896		-10	24	60		Lt superior frontal gyrus

### Correlation between spectral properties (sensor-level) and regional oscillatory intensity (source-level)

MF was positively correlated with delta intensity at the left superior frontal gyrus (Table 1 and Figure 2). No other correlation was found between the spectral properties and regional oscillatory intensity.

## DISCUSSION

The present study revealed four major findings: (i) NPT improved participant cognitive functions as measured by cognitive assessments; (ii) spectral properties of neural oscillatory activity predicted the NPT outcome; (iii) source-level low frequency oscillatory intensity (delta and theta) reflected the current cognitive status; and (iv) source-level high frequency oscillatory intensity (beta and gamma) predicted the effects of NPT on cognitive performance.

### Cognitive data capture nature of NPTs

In the present study, NPT improved participant cognitive performance as measured by both the MMSE-J and the ADAS-J Cog at the group level, and this result is consistent with those of previous studies [9, 14, 25, 26]. The correlation between changes in MMSE-J and ADAS-J Cog scores was positive (*r* = 0.28), although it was not statistically significant (*p* = 0.084). A higher MMSE-J score indicates better cognitive performance, whereas a higher ADAS-J Cog score indicates worsened performance. Thus, the positive correlation between the changes in MMSE-J and ADAS-J Cog scores indicates that the change in each test score detected unique improvements in cognitive functions: some participants showed large improvements in the MMSE-J score, and others showed it in the ADAS-J Cog score. The results suggest that the two assessments are sensitive to different cognitive traits [27]. Indeed, the MMSE-J was developed as a cognitive screening test [28]; in contrast, the ADAS-J Cog was intended to evaluate the severity of cognitive impairment and noncognitive behavioural impairment characteristics of patients with AD [29]. Consequently, the MMSE-J and ADAS-J Cog assessed different aspects of NPT outcome.

Initial cognitive performance partially predicted NPT outcome. Participants with a low MMSE-J ‘Initial Score’ tended to have higher improvements in cognitive performance, although this was not the case with respect to the ADAS-J Cog. The negative association between the MMSE-J ‘Initial Score’ and NPT outcome might be explained in two ways. First, it can be due to the ceiling effect of NPT outcome because participants with a higher initial MMSE-J score have less potential for improving their performance. Second, NPT is more effective for participants with more severe cognitive decline; it is more difficult for participants with a lower MMSE-J score to demonstrate their maximum performance before NPT, although their potential performance is enhanced after the NPT period. Longer periods of NPT and more NPT sessions led to a better NPT outcome based on the ADAS-J Cog. This is in line with a previous study, which showed that a longer NPT period improves its outcome [30]. Frequency of NPT sessions did not exert a positive effect on the outcome, in agreement with a previous study that showed that the frequency and strength of NPT sessions did not have a positive effect on NPT outcome [31, 32]. NPTs, as well as the learning process, are based on neuroplasticity or the ability to modulate neural network efficiency [6, 7]. It is therefore reasonable that NPT takes time to result in significant improvements because ‘there is no royal road to learning’.

### Neural oscillatory activity predicts NPT outcome

Neural oscillatory activity measured using MEG provided four different insights. First, spectral properties of MEG signals predicted improvements in cognitive functions (i.e. ‘Estimated Score Change’ in the present study) rather than the current status (i.e. MMSE-J and ADAS-J Cog ‘Estimated Scores’ in the present study). Previous studies suggested that patients with MCI and AD show distinctive spectral properties of MEG signals: an increase in low frequency oscillations together with a decrease in high frequency oscillations, alpha activity, and irregularity [33–35]. Moreover, these alternations can be quantified by spectral properties of the MEG signals, such as MF, IAF, and SE. The MF represents the spectral power balance between high and low frequency oscillatory activities; it decreases in parallel with the progress of cognitive decline [34–37]. In the present study, a lower MF predicted the improvement in cognitive function when measured using the MMSE-J well, but not when using the ADAS-J Cog. Although this result seems to reflect inconsistency between the two cognitive assessments (MMSE-J and ADAS-J Cog), it is not practical to make further interpretations because of the lack of spatial information. Neither the IAF nor SE predicted changes in cognitive scores. The IAF represents the peak frequency of alpha oscillatory activity, which decreases with the progress of cognitive decline due to AD [38, 39]. The SE quantifies the irregularity of brain activity [40]; thus, cognitively healthy people are characterised by brain activity patterns with high SE values (around 0.80–0.90), which progressively decrease in patients with AD [35–37]. The higher the SE, the more irregular the distribution of oscillatory components. The peak of the PSD around the alpha frequency becomes less prominent with the progression of cognitive decline, and the PSD becomes flat, which is represented by a small SE value [21]. As described above, there are three types of changes in spectral properties according to cognitive impairment (i) power balance between high and low frequency oscillatory activities (MF), (ii) alpha peak frequency (IAF), and (iii) prominence of the alpha oscillatory activity (SE). The sensor-level analysis revealed that the first factor (power balance), rather than change in alpha frequency (IAF and SE), was important to predict NPT outcome. None of the spectral parameters (MF, IAF, and SE) showed statistically significant correlations between the estimated cognitive scores (MMSE-J and ADAS-J Cog) at the time of MEG recording (‘Estimated Scores’). This is in conflict with previous findings that patients with MCI showed enhanced low frequency oscillatory components and low complexity in their neural activity [21, 34, 41], which could be explained by the lack of patients with severe dementia in the present study. The initial cognitive scores were nearly homogeneous; however, pathological brain changes can be heterogeneous, and the severity of pathological changes and cognitive impairments are not correlated [6, 13]. The individual differences in initial pathological changes could have led to the mismatch between cognitive assessment ‘Initial Scores’ and spectral parameters across participants.

Second, low frequency oscillatory intensity (delta and theta) was correlated with present cognitive status. The ADAS-J Cog score was positively correlated with theta intensity at the left angular gyrus, which is a part of the caudal brain. This result could suggest that an increased theta intensity reflected lower cognitive performance. Furthermore, this result is in line with previous findings. Slowing down of spontaneous neural oscillations is observed at the early stage of cognitive decline, and this alteration shifts to the caudal part of the brain with progress of cognitive decline [20, 42–45]. A previous study showed that low frequency oscillatory intensity (2–4 Hz) increased along with the progress of dementia due to AD [20]. The change was prominent in the posterior parietal, occipital, prerolandic, and precuneus cortices, which are located in the caudal part of the brain. Participants enrolled in the present study had MCI or early stage dementia; it is therefore reasonable that enhanced theta intensity was correlated with cognitive decline measured using the ADAS-J Cog (the ‘Estimated Score’). However, the present study also showed that delta intensity at the orbitofrontal gyrus was positively correlated with the MMSE-J score (the ‘Estimated Score’). This result could imply that an enhanced low frequency oscillatory intensity in the rostral brain represents little cognitive decline; this represents a reverse association than that of the previous finding for the caudal brain. Low frequency spontaneous neural oscillations reflect not only cognitive impairments, but also healthy cognitive function, such as memory and attention [46]. Healthy cognitive function enhances low frequency neural oscillations, the so-called “frontal theta”, at the rostral region [47, 48]. The orbitofrontal cortex plays an important role in decision making, which is often impaired in patients with dementia [49, 50]. However, damage to the orbitofrontal cortex does not cause extensive cognitive deficits or problems in daily life activities [51]. All patients enrolled in the present study were living at home and had to make efforts to compensate for their cognitive impairment and to adapt in their daily lives. Thereby, this compensation could have enhanced rostral low frequency neural oscillations; this was clear in participants with a high MMSE-J score because they had the potential to perform healthy cognitive activities for compensation. This idea is further supported by the fact that an increase in spontaneous low frequency oscillatory activity in healthy older adults is associated with better cognitive function [46].

Third, high frequency oscillatory intensity (beta and gamma) was correlated with NPT outcome. Beta intensity at the right post central gyrus, superior frontal gyrus, and middle frontal gyrus was positively correlated with the MMSE-J ‘Estimated Score Change’. High frequency oscillatory intensity is associated with gamma-aminobutyric acid-related neuronal activities [52], which is in turn is associated with neural plasticity [53] and NPTs [6, 7]. Our previous study showed that NPT enhanced beta intensity around the central gyrus, which is consistent with the current results [14]. In addition, we found changes in beta intensity in two other regions: the right superior frontal gyrus and middle frontal gyrus. The superior frontal gyrus contributes to working memory [54]. In this regard, a previous study using functional magnetic resonance imaging (fMRI) showed that patients with MCI and dementia due to AD exhibited more activation than healthy controls in the right superior frontal gyrus during working memory tasks [55]. Another study using structural MRI observed that older adults with low working memory performance had significantly decreased cortical surface area in the right frontal cortex, including the right superior frontal gyrus, when compared with older individuals with higher performance [54]. Working memory deficits also appear in older individuals who are susceptible to cognitive deterioration [56]. The right middle frontal gyrus contributes to attention [57], and attentional impairment is a symptom of MCI [58] and also a factor assessed by the MMSE-J. A similar change was induced by NPT at both the right superior frontal and middle frontal gyri. Therefore, it is reasonable that the change in beta intensity in these regions is correlated with the MMSE-J ‘Estimated Score Change’. Low-gamma intensity at the right angular gyrus was positively correlated with the ADAS-J Cog score (the ‘Last Score’). Although we have already reported that NPT induced low-gamma oscillatory intensity in that brain region, we did not find any correlation with cognitive performance [14]; however, it must be noted that the ADAS-J Cog was not used in the previous study. The present study replicated the findings with a different group of participants at another MEG site with additional information: the change was correlated with the magnitude of change in cognitive performance.

Fourth, predictors of NPT outcome were different between sensor-level (global activity) and source-level (regional activity) analyses. Lower MF and enhanced beta intensity in the right frontal cortex predicted larger cognitive improvement based on the MMSE-J. Higher MF and attenuated low-gamma intensity predicted larger cognitive improvement based on the ADAS-J Cog. It is not easy to interpret the differences between the sensor-level and source level analyses results; these two analyses are complementary to each other, and there was little correlation between the corresponding results (Table 1 and Figure 2). The difference between the two analyses could be explained by the difference in sensitivity to regional brain activity with individual differences: sensor-level analysis can detect two regional activations with a few centimetres of separation due to individual difference because it regards them as an identical activation, while source-level analysis could fail to detect them because it distinguishes them.

### Importance of an objective measurement in clinical situation

In the present study, we predicted the outcome of NPTs using an objective measurement, namely MEG, rather than neuropsychological assessments such as MMSE-J and ADAS-J cog. Neuropsychological assessments are essential tools in the treatment of dementia and they have been widely used and accepted [27]. Although they are extremely useful in general and work well at a group-level, they sometimes face difficulties in clinical situations, which focuses on a single patient level rather than a group. These assessments depend on the skills of clinical psychologists and cannot be properly used for uncooperative patients. At the second or third assessment, the practice effect biases the results, especially for patients with very mild cognitive impairment who remember the last assessment. These limitations cause outliers in the scores. Outliers are not important in a research setting (*i.e.* group level) because the scores are described in statistical terms such as ‘average’ or ‘ SD’. However, they cannot be ignored in the clinical setting where every single patient is important. Objective measurements such as MEG are free from these limitations. For example, few patients cannot remain calm in an MEG shielded room for only 5 min, even if they are not cooperative, and practice effects have little impact on MEG data. Objective measurements are not alternatives to neuropsychological assessments, but rather offer a complementary clinical tool.

### Limitations

There are five methodological limitations that should be considered with regard to the present study. First, cognitive assessments and MEG recordings were conducted on different days because they were carried out at different hospitals during routine diagnostic examinations. Although we estimated the cognitive scores on the days of the MEG recordings, there could have been differences between the estimated and the actual scores. Second, the number of the participants was limited. Although we had initially recruited 22 participants at the beginning of the study, 6 dropped out due to the coronavirus disease outbreak. Fortunately, 16 patients had completed NPT sessions and were happy to continue their participation. Although 16 participants is not a large number, it is generally regarded as acceptable for a neuroimaging study [59]. Furthermore, we used a bootstrapping approach to the statistical testing, which enhanced the stability of the results derived from limited samples. The results were largely in line with those of our previous study [14], which supports the reliability of the present study’s findings. Third, in the present study, only participants with MCI or early stage dementia due to AD, but not those with severe AD or dementia due to other diseases (*e.g.*, Parkinson’s diseases), were recruited. Hence, the present results might not be directly applicable to patients with severe or other types of dementia. Nevertheless, patients with MCI or early stage dementia are the major target populations of NPTs. Thus, our findings can have a significant impact on clinical practice even if the results can be applied only to a limited stage of cognitive impairment. Fourth, canonical MRI images were used for source estimation instead of individual MRI images. This was because MRI scans are often difficult and risky to perform in patients with dementia [60], and the patients are often uncomfortable with an MRI scan due to the loud noises created by the machine. However, in our previous work, we have shown that canonical MRI images are appropriate for group studies on dementia [14]. Fifth, we reported the MMSE-J and ADAS-J Cog scores to show improvement in patients' cognitive performance. Although other neuropsychological scores could be used to make the appropriate diagnoses and monitor patients' condition in more detail, we did not include them in the present study to maintain clarity and focus in our results. We did not include neuropsychological batteries for specific cognitive functions, such as episodic memory, since this was an observational study at a clinical setting, where it is important to minimise the burden to the participants. We performed the additional MEG recordings as part of our standard clinical practice.

## CONCLUSIONS

NPT represents personalised treatment—there are several options, and the optimal strategy depends on the stage of dementia and personality of each patient [11]. NPT is also time-consuming and it generally takes a few months for improvements to be noticeable. However, the patients’ lifetime is limited, especially for older individuals; thus, there is no time for non-effective NPT. If we can predict the outcome of NPT in each individual, the chances of finding the most appropriate therapeutic intervention for each increase. In the present study, we found that the balance between high and low frequency oscillatory activities (MF) predicted the NPT outcome rather than the properties of the alpha oscillatory activity (IAF and SE). More specifically, enhanced high frequency oscillatory intensity at the right hemisphere predicted a positive NPT outcome. MEG scanning is totally non-invasive and only takes 10 min, including 5 min of recording and a few minutes of preparation; thus, MEG can be taken advantage of to find a better NPT strategy for each individual.

## MATERIALS AND METHODS

### Ethics statement

Informed consent has been obtained from all participants and their family members to participate in this study. The investigation has been conducted in accordance with the ethical standards and according to the Declaration of Helsinki and according to national and international guidelines and has been approved by the Ethics Committee of both Mihara Memorial Hospital (#097-01) and Kumagaya General Hospital (#26). Patients and their caregivers were explained that they could decline their participation at any time without giving any reasons, and that this would not lead to any disadvantage in terms of their clinical treatments.

### Participants, NPT procedure, and ethical considerations

This study was conducted as an observational study. Clinical assessments and NPTs were conducted as part of standard clinical practice and additional procedures for research purposes were kept to a minimum. Participants were recruited from patients who were going to undergo NPT at Mihara Memorial Hospital for clinical purposes and met all of the following five criteria: (i) care givers were reliable and healthy, (ii) their clinical dementia rating was lower than 0.5, (iii) activities of daily living score indicated that they required no assistance, (iv) instrumental activities of daily living level indicated that they required some assistance, and (v) they were not receiving and had not received anti-dementia pharmacological treatment. Sixteen participants (10 women; mean age ± SD: 77.3 ± 6.4 years, ranging from 64 to 88 years) with cognitive impairments met these criteria and were enrolled in the present study. Fourteen out of 16 participants were diagnosed with MCI due to AD and one each with early stage dementia due to AD and early stage dementia due to AD with vascular dementia, although they were considered as MCI at the beginning of the study. The diagnoses were made according to the National Institute on Aging-Alzheimer’s Association criteria [61] by two clinicians based on biological and neuropsychological assessments, such as repeated medical interviews, anatomical MRI, single photon emission tomography, blood tests, frontal assessment battery, Frenchay activities index, and life space assessment, as well as the MMSE-J and ADAS-J Cog. One of the two clinicians was a neurologist and member of the Board of Japanese Society of Dementia Research. The other was a neurosurgeon and a clinical instructor of the Japanese Society of Dementia Research. Patients underwent cognitive stimulation therapy as an NPT in small groups (4–6 participants) led by occupational and/or speech therapists for several months (184.6 ± 33.4 days, mean ± SD, ranging from 126 to 245 days). The duration of each NPT session was 120 min/day, and a session was held once a week at most. Required attendance at the NPT session was determined according to each participant’s status after the detailed consultations between the participant, the participant’s family, and hospital staff (*e.g.*, some participants attended NPT sessions every week, while others attended every 2 weeks or every month). The number of NPT sessions was 22.4 ± 4.6 times (ranging from 14 to 29 times). The frequency of the NPT session was 0.12 ± 0.02 times/day (ranging from 0.14 to 0.08 times/day). Cognitive assessment was conducted at both the beginning and the end of NPT, whereas MEG activity was recorded once during the treatment period.

### Cognitive assessments and control definition of variables

Two cognitive instruments were used to evaluate participant cognitive performance: the MMSE-J [28, 62] and the ADAS-J Cog [29, 63–65]. Lower MMSE-J scores and higher ADAS-J Cog scores indicate more severe cognitive impairment. Both types of cognitive assessments (MMSE-J and ADAS-J Cog) were conducted once at the beginning and at the end of the NPT period. The first day of NPT period was defined as the day when participants underwent the NPT for the first time (Figure 1). The initial assessments were made before the first day of the NPT period (MMSE-J: 16.1 ± 11.4 days, ranging from 5 to 42 days; ADAS-J Cog: 8.7 ± 2.9 days, ranging from 5 to 14 days), and the corresponding scores were defined as ‘Initial Scores’. A few months after the first day of NPT, the second set of MMSE-J and ADAS-J Cog assessments were performed on the same day, with the corresponding scores being defined as ‘Last Scores’. The ‘Score Change’ (‘Outcome’) was defined as the difference between the ‘Initial Scores’ and the ‘Last Scores’ for each assessment. In the present study, the ‘NPT period’ was defined as the period beginning from the first day of NPT to the day of the second cognitive assessment (*i.e.* the day on which ‘Last Scores were provided). NPT after the second assessment (*i.e.* the ‘Extra NPT period’ in Figure 1) was ignored. The length of the NPT period was 184.6 ± 33.4 days (mean ± SD, ranging from 126 to 245 days). Resting-state brain activity was recorded for each participant during the NPT period as early as possible. Because there was a considerable delay (34.3 ± 25.7 days, ranging from 0 to 76 days) between the day on which the ‘Initial Scores’ were measured and the day of the MEG recording (*i.e.* ‘MEG delay’ in Figure 1), we estimated cognitive scores at the MEG recording day using a liner model; these were defined as the ‘Estimated Scores’ (Figure 1). In this model, we hypothesised that: (i) the scores did not change between the day on which the ‘Initial Score’ was measured and the first day of NPT, and (ii) the scores changed (*i.e.* improved or worsened) linearly in the NPT period between the first and the last day of NPT. The second hypothesis was supported by a previous study, which showed that the longer the NPT period, the better the outcome [30]. The difference between ‘Estimated Score’ and ‘Last Score’ was defined as the ‘Estimated Score Change’ (‘Estimated Outcome’); it represented the outcome after MEG recording. Two of the 16 participants underwent the MEG scan before the first day of NPT; in this case, the participant’s ‘Estimated Score’ was considered the same as the ‘Initial Score’. Eleven of the remaining 15 participants underwent MEG scans before completing 30% of their NPT period, whereas MEG acquisitions for the rest of the participants were carried out later.

To assess the improvement in cognitive performance, the ‘Score Change’ (‘Outcome’) was tested for the null hypothesis (with the values being equal to 0) using a one-sample *t* test. Considering the relatively small number of participants, an equivalent bootstrapping approach was used. The average ‘Score Change’ was computed by resampling with replacement data across all participants 20,000 times, and the percentage of the resampled average data, being larger or smaller than 0 (the smaller value), was taken as the significance level (*p*-value).

### MEG scanning

All participants visited the MEG centre at Kumagaya General Hospital, Japan for measurements of their spontaneous neural oscillations (*i.e.* resting-state brain activity) using MEG during the NPT period as early as possible. Spontaneous neural oscillations were recorded for 5 min using a 160-channel whole-head type MEG system (RICOH160-1; RICOH, Tokyo, Japan) in a magnetically shielded room. During the scan, participants were asked to remain calm in the supine position with their eyes closed. The scanning conditions were controlled to be as consistent and comfortable for participants as possible. The sensor and reference coils were gradiometers 15.5 mm in diameter and 50 mm at the baseline, and each pair of sensor coils was separated by a distance of 23 mm. The sampling frequency was 2,000 Hz with 500 Hz low-pass filtering during the recording. To co-register MEG source images with structural brain images acquired using canonical MRI, three fiducial magnetic marker coils were placed on each participant’s face (5 mm above the nasion and bilaterally 10 mm in front of the tragus) during the MEG scan.

### MEG data analysis

**Figure 3:**
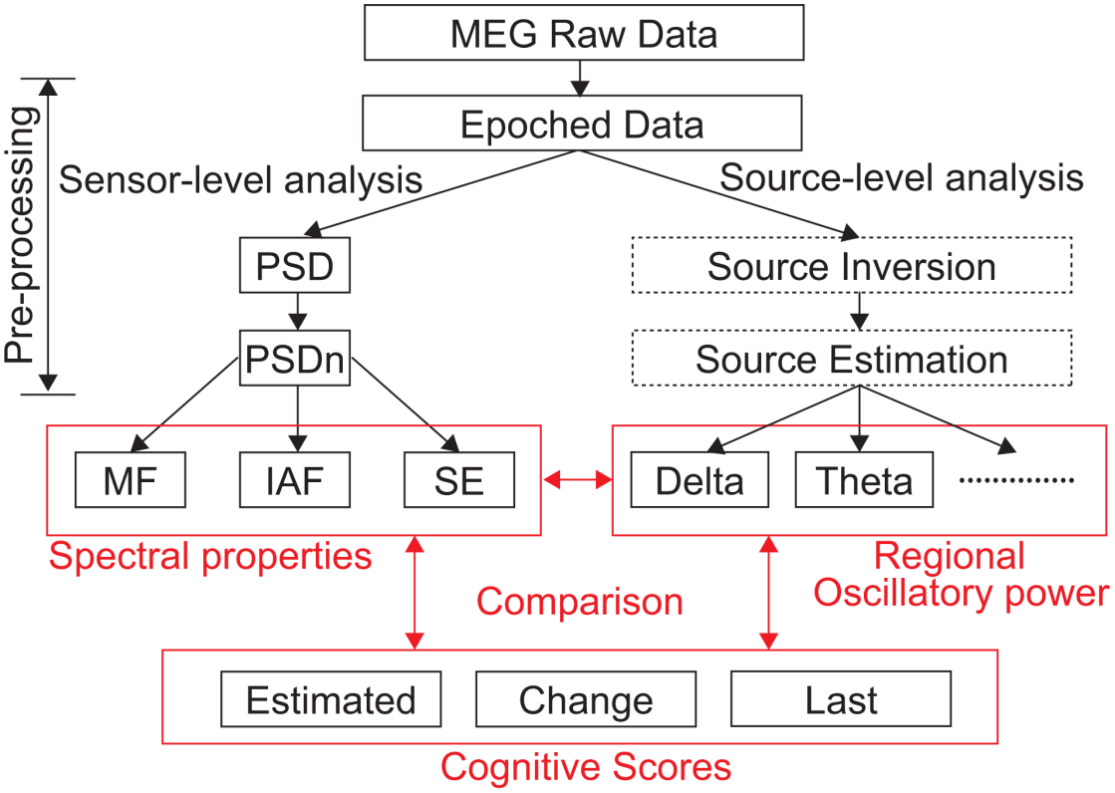
**Data analysis pipeline.** PSD, power spectral density; PSDn, normalised PSD; MF, median frequency; IAF, individual alpha frequency; SE, Shannon spectral entropy; Estimated, Estimated Score; Change, Estimated Score Change; Last, Last Score; MEG, magnetoencephalography.

MEG data were pre-processed offline using the software package SPM-12 (Wellcome Trust Centre for Neuroimaging, London, UK; https://www.fil.ion.ucl.ac.uk/spm/) and the MEAW system (https://www.hokuto7.or.jp/hospital/lang/english-home/meaw/). Two types of standard MEG analyses were applied: sensor-level and source-level analyses (Figure 3). Each of them has some advantages and drawbacks. The sensor-level results are mathematically reliable and are useful for identifying three types of neural changes: (i) enhanced low frequency oscillatory activity accompanied by attenuated high frequency oscillatory activity, (ii) slowing down of the alpha peak frequency, and (iii) less prominent alpha oscillations. It is advantageous to find global changes rather than regional ones; however, biological/medical interpretations are difficult due to lack of accurate information about brain regions. In this regard, source-level analysis has rich biological/medical implications because it provides regional information about the brain. The potential of this kind of analysis to predict the NPT outcome was demonstrated in our previous work in which we reported its sensitivity to detect subtle changes in oscillatory neural activity due to NPTs [14]. Nonetheless, source-level results can be unstable (*i.e.* algorithm-dependent) and less sensitive to changes with large inter-individual differences at the regional level. Therefore, we applied both approaches (*i.e.* sensor- and source-level analyses) to obtain an accurate and thorough characterisation of the neural changes associated with NPT.

### Sensor-level data processing

In the case of sensor-level analysis, artefacts were manually removed by principal component analysis, if necessary, using the analysis software provided by the MEG manufacturer because spectral parameters are sensitive to artefacts. A 50-Hz band-stop filter was applied to remove power line noise. Thereafter, three spectral parameters were calculated to summarise different properties of spontaneous neural oscillations: MF, IAF, and SE [36]. These were computed from the power spectral density (PSD), which was estimated using the Blackman–Tukey method considering non-overlapping 10-s segments. Afterwards, the PSD was normalised between 1 and 70 Hz (PSDn) [66]. The first parameter, MF, is the median of the distribution represented by the PSDn (*i.e.* the frequency that splits the PSDn into two halves of equal power). It has demonstrated its usefulness to quantify the slowing of spontaneous neural oscillations in patients with dementia [35], which reflects the increase of low frequency oscillatory components along with the decrease of high frequency neural activity in patients with cognitive impairment. The second parameter, IAF, is the frequency corresponding to the peak of the PSDn in the alpha band (*i.e.* the dominant alpha activity), which usually appears in human adults in the eyes-closed resting condition. IAF is useful for describing the loss of neural oscillations at the alpha band (*i.e.* the ‘shift-to-the-left’ of the alpha peak), which is commonly observed in dementia [35]. Finally, SE is an irregularity measure closely related to the concept of order in information theory, which quantifies the distribution of the oscillatory components of the PSDn. The SE has shown its usefulness to quantify the loss of irregularity associated with the less prominent alpha oscillations in patients with dementia [35]. To test the hypothesis that these three spectral parameters predict the NPT outcome, we examined the pairwise associations between the following factors: participant age, NPT information (A: total days in the NPT period; B: number of NPT sessions attended; and C: frequency of the NPT session = B divided by A), cognitive assessment scores (MMSE-J and ADAS-J Cog ‘Initial Score’, ‘Last Score’, ‘Estimated Score’, ‘Score Change’, and ‘Estimated Score Change’), and spectral parameters computed from sensor-level MEG data (MF, IAF, and SE). As with the analysis for cognitive assessment, a bootstrapping approach was used to evaluate the correlations. For each pair of variables, Pearson’s coefficient was calculated by resampling with replacement data across all participants 20,000 times. The percentage of the resampled coefficients, being larger or smaller than 0 (the smaller value), was taken as the significance level (*p*-value). The false detection rate was controlled using the Benjamini and Hochberg method [67].

### Source-level analysis

The source-level analysis procedure followed the pipeline used in a previous study [14] (Figure 3). The continuous MEG signals were divided into non-overlapping 10-s segments. Because the experimental environment generated a utility frequency, a 50-Hz band-stop filter was applied to the epoched data. These filtered data were then directly used for source-level analyses. To identify the brain regions producing the resting-state-induced components, the source inversion procedure was applied to the delta (0–3 Hz), theta (4–7 Hz), alpha (8–12 Hz), beta (13–25 Hz), and gamma (low-gamma, 26–40 Hz; high gamma, 41–80 Hz) oscillatory components separately, using a maximal smoothness algorithm with a spatially coherent sources model (*i.e.* the COH algorithm implemented in SPM-12) [68], which is comparable to standardised low-resolution brain electromagnetic tomography [69]. The COH algorithm is a popular source inversion algorithm and is often used in clinical environments [70, 71]. Forward modelling was performed for the whole brain using a single shell model with canonical MRIs provided by SPM-12. The source inversion and estimation were performed by applying filters corresponding to each frequency band (from delta to high gamma). No source priors were used for source estimation. The estimated oscillatory intensity at each frequency band at each brain region (*i.e.* regional oscillatory intensity) was saved as a source image file in the NIfTI format. The source images were smoothed (20 × 20 × 20 mm) and used for the second (group)-level analysis. Three types of second (group)-level analyses were performed to find: (i) brain regions in which oscillatory intensity was correlated with the cognitive scores at the scanning day (‘Estimated Score’), (ii) brain regions in which oscillatory intensity was correlated with the ‘Estimated Score Change’, and (3) brain regions in which oscillatory intensity was correlated with the cognitive scores at the end of the NPT period (‘Last Score’) (Figure 1). The source images were regressed according to the MMSE-J and ADAS-J Cog scores separately in the three ways described above. Both positive and negative effects of predictors were evaluated by building *t*-contrasts with +1 and −1. Here, we report the source locations of peak level activations at a significance threshold of *p* = 0.05 (corrected for family-wise error rate) and a cluster extent at *k* > 10 (= 80 mm^3^) [72]. Cortical areas at which the peaks of the estimated sources were located were identified using SPM-12.

### Data availability

Data are available from Shigihara, Yoshihito, 2020, ‘NPT for MCI’, https://doi.org/10.7910/DVN/P2SJPA, Harvard Dataverse.
